# In a green frame of mind: perspectives on the behavioural ecology and cognitive nature of plants

**DOI:** 10.1093/aobpla/plu075

**Published:** 2014-11-21

**Authors:** Monica Gagliano

**Affiliations:** Centre for Evolutionary Biology, School of Animal Biology, University of Western Australia, Crawley, Western Australia 6009, Australia

**Keywords:** Affordances, agency, consciousness, decision-making, kin selection, learning, memory, perception.

## Abstract

It is increasingly recognized that plants are highly sensitive organisms that perceive, assess, learn, remember, resolve problems, make decisions and communicate with each other by actively acquiring information from their environment. However, the fact that many of the sophisticated behaviours plants exhibit reveal cognitive competences, which are generally attributed to humans and some non-human animals, has remained unappreciated. Here, I will outline the theoretical barriers that have precluded the opportunity to experimentally test such behavioural/cognitive phenomena in plants. I will then suggest concrete alternative approaches to cognition by highlighting how (i) the environment offers a multitude of opportunities for decision-making and action and makes behaviours possible, rather than causing them; (ii) perception in itself is action in the form of a continuous flow of information; (iii) *all* living organisms viewed within this context become agents endowed with autonomy rather than objects in a mechanistically conceived world. These viewpoints, combined with recent evidence, may contribute to move the entire field towards an integrated study of cognitive biology.

## Abstract (precis)

It is increasingly recognized that plants are highly sensitive organisms able to perceive, assess, learn, remember, resolve problems, make decisions and communicate with each other by actively acquiring information from their environment. If plants so efficiently control the way they interact and behave in their environment, would it be appropriate to say that they have a mind of their own? In this paper, I propose that the time is ripe for asking this and related questions about the mind of plants and suggest how we may venture into this unexplored mindscape.

## Perception and Cognition as an Evolutionary Essential Feature of Living Systems

Everything any living organism knows about the world comes to it through its senses. Such a deceptively simple task bears the most crucial challenge faced by all organisms—the requirement to use a diversity of sensory organs and signal-transduction systems (i.e. stimulus–response pathways, Clark *et al.* 2001) to sense the surrounding environment and ensure the most appropriate adaptive responses in order to survive and proliferate in a range of ecological niches. The total process of receiving, organizing and interpreting such an enormous variety of inputs culminates into what is generally referred to as perception. Perception fundamentally shapes the choices, decisions and actions organisms take, and hence it is an essential feature of living. Evolutionarily, a close match between perception and reality is advantageous as it allows for the gain of accurate information about a dynamic world filled with potential dangers, where small mistakes can sometimes have fatal consequences. A stark demonstration of the importance of correctly matching perception with reality can be seen whenever we negotiate the morning traffic on the way to work by timely and accurately braking and steering our cars; but of course, it underpins all interactions organisms experience in their environment, whether they are looking for shelter, finding food, avoiding predators, securing mates and so on. Paradoxically, information about the world is virtually always misperceived because an organism's past experiences and its expectations of the future unescapably colour the perception of its current reality, a reminder that each organism ultimately exists in its subjective perceptual world (i.e. the Uexkullian notion of ‘Umwelt’; see Von Uexkull 1934/1957). That being said, the mismatch between reality and the perception of it is opportunely remedied by the very cognitive components (e.g. memory, learning, decision-making) that influence the way an organism perceives the external world. The existence of this continual interaction of perceptual and cognitive abilities emphasizes that there may be no sharp division between the two systems (to the extent that some researchers even question the significance of distinguishing between the two systems from the onset; see Tacca 2011; Cahen and Tacca 2013).

Over the last 25 years, the relevance of cognitive psychology to behavioural ecology, and more explicitly, the role that cognition plays in the production of many behaviours within the other-than-human domain, has received increasingly growing consideration (e.g. Yoerg 1991; Shettleworth 2001; Calvo and Keijzer 2009). By integrating psychological and biological approaches to the studies of cognition beyond the human sphere, research in numerical cognition, for example, has shown that several other species across taxa are able to count and master a variety of numerical competences from numerical discrimination, ordinal abilities to simple arithmetic (see Davis and Perusse 1988; Brannon and Roitman 2003; Shaun *et al.* 2010), which are useful in mating strategies, navigation, foraging and visual decision-making (e.g. Dacke and Srinivasan 2008; Vallortigara *et al.* 2010; Bar-Shai *et al.* 2011; Carazo *et al.* 2012; Nelson and Jackson 2012). Similarly, there is now extensive experimental evidence that social learning, for example, plays an important role in the development of behaviour in a wide range of taxonomic groups, including mammals, birds, fishes, insects (Brown and Laland 2003; Leadbeater and Chittka 2007; Hoppitt and Laland 2008; Thornton and Clutton-Brock 2011; Guttridge *et al.* 2013) and recently, implicated in plants too (Baluška and Mancuso 2007; Gershenzon 2007). In contrast to asocial learning (e.g. trial and error), learning by observing or interacting with others can offer a cheap way of acquiring valuable information about the world (Heyes 1994; Rendell *et al.* 2011; see also discussion by Laland 2004). In effect, it has major ecological and evolutionary implications by mediating, for example, collective behaviour that enables a group of individuals to solve cognitive problems that go beyond the capacity of the single individual (i.e. swarm intelligence [SI]; in animals, see review by Krause *et al.* 2010; in plants, see Baluška *et al.* 2010; Ciszak *et al.* 2012), facilitating altruistic behaviour towards familiar individuals through kin recognition (e.g. Komdeur and Hatchwell 1999; Tang-Martinez 2001; Dudley and File 2007; Frommen *et al.* 2007; Villavicencio *et al.* 2009), and more generally, promoting cooperation within a group of individuals with the associated benefits of greater detection of predators, access to better quality resources, greater survival of young and more (e.g. Simard *et al.* 1997; West *et al.* 2002; Hayes *et al.* 2009; Murphy and Dudley 2009; Beckerman *et al.* 2011; Falik *et al.* 2011). Altogether, it would be surprising not to find organisms equipped with mechanisms adapted to perceive a variety of forms of sensory inputs from the surrounding world (i.e. the perceptual system), transduce them into a common signal that punctually activates different parts of the body (i.e. the cognitive system) to produce an output of precise actions and the associated behavioural displays we see in all biological organisms. Then the key challenge is to venture across the traditional taxonomic boundary and beyond the animal realm, to reveal the biophysical and physiological mechanisms mediating this process of ‘translation’ and to explore the phylogenetic diversity of these mechanisms within a single theoretical framework.

In this Point of View, I propose that the time is ripe for a systematic investigation of the cognitive capacity of plants. Specifically in the following paragraphs, I aim to (i) outline the current theoretical difficulties associated with the study of cognition in non-human organisms (including plants) and propose alternative approaches to cognitive research and (ii) review the existing evidence for cognition in plants, showcasing some recent examples in plants as starting points for applying a more integrated approach to the study of cognitive biology across taxa.

## Theoretical Benchmarks for the Study of Plant Cognition

Because of its traditional foundation in human psychology, the modern study of cognition assumes, to a greater or lesser extent, that human cognitive abilities constitute the standard template for theorizing on the issue. This reasoning predominantly rests on the premise that the brain and a neural system are required to realize the complex computational processing that enables faculties such as anticipation, awareness, memory, self-reference, motivation, decision-making, learning, communication and more, which are, broadly speaking, attributes of what we call, the mind. Taking human cognition as the diagnostic reference point to investigate what cognitive features are present in non-human others is inescapably anthropocentric and confines the interpretation of reality they experience solely in terms of human values and perception (i.e. anthropomorphism). In our own defence, the ascription of human qualities and mental states to non-human others may not simply be an inveterate habit of ours (e.g. personification of animals, natural phenomena or deities over millennia of storytelling), but a trait inherently ‘wired’ in our biology (Press 2011). Neuroimaging studies, for example, have shown that humans respond more strongly to the observation of human, rather than non-human movement (Oberman *et al.* 2007). Interestingly, however, the observation of humanoid robots (which are built to resemble the human body) can activate the same response in our neuronal system, a sign that our brains (literally) cannot help but assign human attributes to others when they resemble human actions (Gazzola *et al.* 2007). What these studies reveal is that our understanding of the behavioural and cognitive features of non-human others is at least partly tied up with our own perception of movement. Unfortunately, this instinctive connection between cognition and human-like movement excludes species that also accomplish these feats but in completely different ways. In other words, the critical issue here is that a theoretical construct resulting from this operational stance is sure to judge the behaviour of other species subjectively and, most importantly, deny the presence of cognitive abilities which others (e.g. non-neural and presumably motionless organisms like plants) possess and apply to solve problems and make a living (see Griffin 1976 and Warwick 2000 for discussions on this topic).

One way to move beyond our anthropocentric tendencies is to approach cognition from a wider biological perspective. One such perspective on cognition was offered by the Chilean biologist Humberto Maturana, who suggested that organisms could be viewed as intrinsically part of the environmental niche with which they interact and the niche itself can be understood as being determined by the living system that specifies it (Maturana 1970/1980). According to Maturana's viewpoint, the domain of these interactions is the cognitive domain and cognition is the organization of actual functions and behaviours that make a range of interactions possible and maintain the continuous and uninterrupted production of further interactions. From this perspective, cognition is not a fixed ‘property’ of an organism but rather a dynamic ‘process’ of interactions in the organism–environment system. By viewing cognition as a natural biological phenomenon contributing to the persistence of organisms in constantly changing environments, it then makes sense to approach cognition in human as well as non-human others like plants, as a functional process understood in the context of phylogenetic continuity (see ‘the biogenic approach’, Lyon 2005). Viewed through this lens, cognition does not equate with the presence of a nervous system; the nervous system may expand an organism's range of potential actions and interactions but does not in itself generate cognition. With a nervous system or not, the presence of cognition and the array of cognitive capacities in living organisms may be understood as the workings of a continuous process of evolution by natural selection (Lyon 2005), hence advocating a paradigm capable of unifying a great diversity of expressions of the raw cognitive foundation common to all living systems.

## Existing Evidence for Cognition in Plants

The proximate and ultimate mechanisms used by animals to sense their environment, learn from it and share this information by communicating with each other have long been the subject of intense scientific interest. It is now abundantly evident that animal behaviour is more sophisticated than we have ever thought and that even simple reflexes (sometimes still referred to as ‘noncognitive’) can result in the complex and flexible cognitive structures we refer to as ‘higher learning’ (Shettleworth 2001). In plants, behavioural research exists, yet is not as advanced and recognized. Generally speaking, plant behaviour is still assumed to be rather rigid, stereotyped and inflexible, and even when plants demonstrate cognitive competences such as the ability to learn, for example, their learning capacity is widely considered to be fully pre-programmed. While the cognitive mechanisms in plants are still to be identified, new evidence for plant cognition is enticing and suggests that plants may be far more sophisticated than we had originally imagined.

Over recent years, experimental evidence for the cognitive nature of plants has grown rapidly (e.g. Runyon *et al.* 2006; Karban and Shiojiri 2009; Murphy and Dudley 2009; Broz *et al.* 2010; Heil and Karban 2010; Bastien *et al.* 2013; Dudley *et al.* 2013; Gagliano *et al.* 2014; Gianoli and Carrasco-Urra 2014; Semchenko *et al.* 2014 and many more). It has revealed the extent to which plant perceptual awareness of environmental information directs behavioural expressions and highlighted how many of these behavioural feats and associated cognitive abilities are, in fact, pretty easy to observe. The study by Gagliano *et al.* (2014), for example, primarily concentrated on habituation as a measure of learning capacities in *Mimosa pudica*, demonstrating perceptual awareness, learned behaviours and memory in this plant. Other recent studies, such as by Dudley and File (2007) and Karban *et al.* (2013) for some examples, have elegantly demonstrated the ability of plants to assess relatedness, recognize and discriminate between kin and non-kin both above- and belowground, and exhibit differential treatments of conspecifics based on cues that vary with such level of relatedness (reviewed by Biedrzycki and Bais 2010). In some species, we know that the selective avoidance of wasteful competitive interactions, for example, does occur between genetically identical individuals (e.g. Holzapfel and Alpert 2003; Gruntman and Novoplansky 2004) as well as genetically different but closely related individuals (e.g. Dudley and File 2007). Moreover, by showing that plants receiving the volatile emission cues from self-cuttings were damaged less than plants that were signalled by non-self-cuttings. The study by Karban and Shiojiri (2009) demonstrated a tangible benefit for plants interacting with kin versus non-kin plants, indicating a clear evolutionary trade-off in plant kin selection.

In all cases, to adjust underground root placement or aboveground plant height in response to the presence of neighbours, for instance, neighbour perception alone is not enough to ensure the most appropriate adaptive response in order to survive (see review by Novoplansky 2009). Because the appropriateness of a response depends on the prevailing circumstances and expected future interactions, plants must be able to establish where they are in the context of their physical environment and in relation to other organisms. While many important aspects of how plants may achieve this still remain little understood, the fact is that plants, like animals, certainly have such ‘sense of place’ and an awareness of the neighbourhood they occur in (e.g. Gagliano *et al.* 2012*a*; Gagliano and Renton 2013). Several studies have demonstrated that plants are able to orientate themselves by sourcing their information via both internal body-centred (idiothetic) cues, such as proprioception and body posture (e.g. Bastien *et al.* 2013), and external (allothetic) cues. Specifically, the external cues can arise from spatial elements present in the physical environment (e.g. sunlight; belowground obstructions, Semchenko *et al.* 2008), as well as from the presence of other organisms sharing that environment, including how these others look (e.g. mimicry, Gianoli and Carrasco-Urra 2014) and smell (e.g. volatile emissions, Karban *et al.* 2014), the noise they make (e.g. sounds and vibrations of various kinds, Gagliano *et al.* 2012*b*; Appel and Cocroft 2014) as well as their direct (e.g. Semchenko *et al.* 2007) or indirect physical contact (e.g. Simard *et al.* 1997; Babikova *et al.* 2013). In animals, there is little doubt that awareness of one's position and orientation in space is essential for avoiding obstacles, finding food while avoiding predators, locating potential mates, defending old territories as well as seizing new ones, and this is considered among the most fundamental cognitive processes required for survival (Kimchi and Terkel 2002). The examples above together with numerous findings that keep emerging in the scientific literature on the topic clearly indicate that this is also true for plants. I propose that the cognitive processes involved in the life of plants have not been explored to anywhere near their full potential, leaving serious gaps in our current understanding of the behavioural and cognitive complexity of these organisms.

## Towards an Integrated Approach to Cognition

Given the numerous examples provided here, that plants are cognitive organisms need not be in question. What we should really be asking is how plants, like any other organism whether human, animal or microbe, exhibit and make good use of their cognitive capacities in their life (and how we may observe them). I propose that exploring the cognitive domain in terms of a dynamic process of interactions in the organism–environment system (as suggested by Maturana 1970/1980) may offer an effective and integrated way to approach cognition. How shall we go about doing this? Let us start by considering perception, for instance, as the experience of making contact with the world and exploring what opportunities the environment has on offer. The experience of what opportunities are ‘afforded’ by a given environment (also referred to as ‘affordances’; Gibson 1977, 1979) may take many different forms but it is an intrinsic and fundamental feature shared by all living organisms. Through this process of discovery and dynamic appraisal of the multiple opportunities presented to an organism, the environment facilitates cognitive responses such as prediction and anticipation, and enables an organism to know about the state of the world before deciding and acting in it.

Because affordances are real and perceivable features of the whole organism–environment system (Chemero 2008), this is an ecological theory that offers a much needed practical approach to the study of perception, cognitive abilities and behaviours across all taxa. Its principles have already been effectively applied in various contexts from the importance of body-scaled information for affordances in relation to human movement (e.g. Warren 1984; Warren and Whang 1987), to the essential role of learning about the functional affordances of a task or a tool for solving problems (e.g. birds, von Bayern *et al.* 2009; monkeys, Nelson *et al.* 2011). And more recently, a study on the ability of locusts to perceive affordances when negotiating obstacles in their environment, for example, has shown how an accurate estimate of the insect's own physical characteristics (i.e. self body-size perception) enables it to assess the relative size of the obstacle, to decide whether or not it is passable, and, based on that evaluation, coordinate its attempt to overcome it (Ben-Nun *et al.* 2013).

The concept of affordances has also been adopted within other theoretical frameworks (see the Tau Theory, Lee 1976; see also discussion by Fajen 2007) developed to better understand the coordination of visually guided actions and explain how, for example, we break to stop our car (e.g. Lee 1976) or how pilots and birds do what they do during flight control and landing (e.g. Lee *et al.* 1991, 1993; Padfield 2011). It has also provided a new appreciation for how echolocating bats use acoustic information for in flight guidance to steer themselves to a destination (Lee *et al.* 1992, 1995). I believe that these concepts and approaches can be easily incorporated to enhance and develop our understanding of the behavioural and cognitive ecology of plants. In the following paragraphs, I will offer two analogies as examples illustrating the possible directions to test this.

### Example 1—Orientating in 3D space

As mentioned in the previous section, we now know that plants are, for example, sensitive to the soundscapes that surrounds them and, most importantly, are capable of emitting their own clicking sounds as well as detecting acoustic signals from others (Gagliano *et al.* 2012*b*; Appel and Cocroft 2014). It is conceivable that a plant, like an echolocating bat, could emit sonic clicks and ‘listen’ to their returning echoes allowing it to attain information about its surrounding environment and the neighbourhood contained in it (M. Gagliano, unpubl. data). Echolocation as a form of self-communication (Bradbury and Vehrencamp 1998) could be an efficient way for plants like twiners and tendril climbers to wend their way in the 3D space, track moving objects as well as detect stationary obstacles and, most importantly, locate suitable host trees or other scaffolds to climb on to or attach to. In the case of the latter, supports of different materials and structural qualities are expected to reflect or absorb an incoming acoustic wave in different ways, hence determining the degree and clarity of echoes bouncing back and the perceived affordance a given structure provides to the plant. Naturally, this would allow the plant to make the appropriate behavioural and/or physiological decision within the context.

### Example 2—Echolocating the neighbourhood

As different plant species produce different acoustic emissions (M. Gagliano, unpubl. data), it is plausible to consider that plants may exploit species-specific sounds to characterize who is growing next to them, as we know plants do with light signals bouncing off their neighbours (Aphalo *et al.* 1999; Collins and Wein 2000). In the animal literature, it has become increasingly apparent that echolocating bats, for example, are listening for echoes not only for orientation during foraging and navigation, but also for characterizing their neighbourhood and discriminating between familiar and unfamiliar individuals (e.g. Voigt-Heucke *et al.* 2010). Given the growing evidence for kin selection in plants (see examples in the previous section), this has the potential to open a brand-new and exciting direction for future plant research. Of course, the field of plant bioacoustics is still at its infancy and these ideas are clearly highly speculative as no experimental evidence is available to support them at this stage; yet at risk of overreaching, I would invite the readers to remain nevertheless open to consider such possibilities.

## Concluding Remarks

By revealing a level of complexity in behaviours previously thought to be the exclusive domain of animals, scientific evidence over the last couple of decades has strongly challenged the Aristotelian view that the divide between plants and animals is the absence of behaviour in the first, and the presence of behaviour in the latter and demanded a revised definition of behaviour to include plants (e.g. Silvertown and Gordon 1989; Silvertown 1998). Described as a response to environmental stimuli within the lifetime of an individual, such a definition certainly succeeds in including plants in the behavioural realm but still restricts their responses to simple signal-induced phenotypic plasticity (as previously discussed by other authors, who have clearly pointed out the problems with equating plant behaviour only with plasticity; see Karban 2008; Trewavas 2009 for great examples). By fundamentally retaining unaltered the attitude that plants only react instinctively in a stereotyped and predetermined way, the new formulation inherently lacks in the two ingredients that ‘make’ behaviour: ‘action’ and ‘agency’. Indeed when considered in animals including humans, behaviour generally implies movement (action) and cognitive capacity (agency). Currently, this consideration is not usually extended to plants because evidence for both action and agency has gone undetected (until the recent advent of advanced high-speed cameras, for example, allowing us to shift our perceptual range into one that relates to plants; e.g. Vincent *et al.* 2011) or was simply assumed to be absent.

In my opinion, it is this restricted perspective that has precluded the opportunity to experimentally test such behavioural/cognitive phenomena in plants, until recently. In this Point of View, I have attempted to present a more open interpretation of cognition, fundamentally based on Humberto Maturana's biology of cognition and James Gibson's ecological psychology as well as many others that followed them. The main points may be summarized as: (i) by uninterruptedly offering a multitude of opportunities for decision-making and action, the environment *invites* actions and makes behaviours possible, rather than causing them; (ii) by providing a continuous flow of information, perception in itself is *action* and constitutes one of the two important ingredients that ‘make’ behaviour, as mentioned above; and (iii) *all* living organisms viewed within this context become agents endowed with autonomy rather than objects in a mechanistically conceived world (see a recent review and an in-depth discussion on the topic by Withagen *et al.* 2012).

Finally, I have highlighted the wealth of information already accessible to us in the hope that we may not shy away from the study of plant cognition, but rather we feel inspired to approach it in the context of a unified view of behavioural ecology.

## Sources of Funding

The work is supported by an Australian Research Council Discovery Early Career Researcher Award and Early Career Fellowship Support Program of University of Western Australia.

## Conflicts of Interest Statement

None declared.
